# Toward the way forward: building an emergency mental health system for Israel

**DOI:** 10.1186/s13584-015-0038-3

**Published:** 2015-09-15

**Authors:** Merritt D. Schreiber

**Affiliations:** Center for Disaster Medical Sciences, Department of Emergency Medicine, University of California Irvine School of Medicine/UCI Medical Center, Orange, CA USA

## Abstract

A number of related changes have evolved over the past 25 years: the development of a truly national disaster mental health service in Israel; progress in the science of risk, resilience and evidence base care for those suffering from traumatic stress related disorders; and the development of conceptual models of population level disaster mental health response in the context of emergency management systems such as the Incident Command System.

In a recent IJHPR article, Bodas, et al. report on the dynamic history of disaster mental health response in Israel, which informed by the all too numerous real world events affecting the region. What is most striking is that the system now in place reflects true “lessons learned” in that problems and issues identified in incidents informed deliberative planning, and the current system reflects many iterations of “lessons observed and learned”. There appears to be commitment across sectors of government in Israel that the mental health consequences of disasters and terrorism are important and a priority. This is advanced thinking and sound policy.

As the system in Israel continues to evolve, additional possibilities are offered for further consideration, based on the author’s US-centric experience, to advance emergency response systems in Israel, the Middle East and around the world.

## Background

The recent IJHPR paper by Bodas and colleagues [1] provides a detailed overview of Israel’s national efforts to develop an emergency mental health system, in light of a series of armed conflicts and terror attacks.

Since it began in the 1980’s, the emergency mental health system in Israel has evidenced considerable change and growth in terms of service delivery settings, coordination, payor/funding mechanisms, scope of services and levels of care. These authors describe in detail how these changes have been influenced by real world events, including the threat of ballistic missile attack involving chemical weapons during the first Gulf War. Other changes were seemingly influenced by changes in organizational structures, distribution of responsibility between local communities and various components of the national government. In its current formation, the system appears to provide a multi-tiered response, focused on on-site services by local teams, support centers that provide services to those presenting, and resilience promotion activities via NGOs and other settings.

This commentary will consider the following aspects of the evolution of the emergency mental health system in Israel: process of change, funding, models and range of services, locations, and timing.

## Process of change

What is particularly fascinating to a non-Israeli reader is how the model has been significantly influenced by numerous real world events and how those responsible for the emergency mental health system have adopted a true “lessons learned” approach. For example, following the deployment of the Iron Dome missile defense system, a reduction in direct help seeking was observed, alongside an increase in so called “hotline” use. This required training of the call takers to identify those at higher risk for serious psychological consequences, and such training was then duly provided. Indeed, among numerous innovations, perhaps the most notable is the process innovation of adapting the national emergency mental health system following large scale events.

In many countries, there are “after action” reports and/or large scale recommendations for change. Yet the impact of these after action reports and commission reports is often hard to see in larger countries. Some of the changes in Israel reported by Bodas and colleagues have been sustained and others determined to be less so, so that this candid report is also strikingly refreshing and perhaps unique. In Israel, there seems to be deliberative planning that takes into account issues that truly become lessons learned that produce change and not merely ‘lessons observed’.

### Funding

Once the emergency mental health planners in the United States and elsewhere recognized the need to go beyond single session debriefing for trauma victims and to include longer, evidence based, interventions (such as Trauma Focused Cognitive Behavioral Therapy), the need to secure special funding for these services became evident. Timely trauma care can provide substantial benefits to the individual, their families and society as a whole, as been observed with timely care for depression in the United States [2]. However, the planners identified that current insurance protocols made financial access to care an additional burden on families, preventing timely use of trauma care . In Israel, the funding scheme for disasters has evolved over time to increasingly make access to care possible through novel partnerships with the single payer health insurance system, NGOs and other partners.

In the United States, since the late 1970s, a novel federal disaster mental health program provides financial support to states following a presidentially declared disaster and is known as the Crisis Counseling Program. However, this program does not typically provide for professional care and those needing professional care after a disaster still face significant financial burdens. Following 9/11, an extension of the Crisis Counseling program called “Enhanced CCP Services”, and more recently the “Specialized Crisis Counseling Services Program”, has expanded on the original non-professional services model and provided a larger array of services. Research on these expanded CCP services found beneficial effects [3], and suggested their broader expansion [4], http://www.phe.gov/Preparedness/legal/boards/nprsb/Documents/nsbs-dmhreport-final.pdf). It is not year clear to what extent the US Affordability Care Act (ACA) will also address financial barriers to trauma care. In the US, the major NGO providing disaster mental health services, the American Red Cross, does not typically provide definitive care and services are restricting to 2 crisis intervention sessions and up to 2 follow up sessions. Solving the payer issue to facilitate access to evidence based care remains a challenge.

## Services

The article by Bodas and colleagues describes various changes over time in the provision of types and levels of care. For example, in the 1980’s, before research on the lack of salutatory effects of single session debriefing became known, that approach was widely utilized, along with undefined “mental health aid”. Following these findings, other approaches, including notably CBT, have been increasingly deployed. What is not clear however is the extent to which even more recent findings of meta-analyses [5] and consensus reports [6] have been incorporated. These have found, for example, that Prolonged Exposure CBT has the best evidence of outcome [6] in adults and in the case of children, Trauma Focused CBT [7, 8] has the best evidence of outcome. In fact, the provision of services of any kind to children are not mentioned in the Bodas et al. paper and because children may be the single highest risk age group [3], there is a need to urgently address the needs specifically of children. Interestingly, novel disaster models for services to children have been developed in Israel, but it is not clear if they are being used in the model currently employed [9] and it is unclear whether a national strategy for the needs of children is operative.

Another known population with significant natural resilience, but also elevated risk, is the responder population. It is not clear from the Bodas et al. article how they are served in the Israeli emergency mental health system—either in services provided by the Home Front Command or elsewhere. This may be a gap requiring further work. In the US, a model known as ‘Anticipate. Plan. and Deter: building responder resilience’ is one example of a model for providing a comprehensive, stepped care approach to responders and health care workers in particular (https://iab.gov/Uploads/IAB%20Stress-Related%20Mental%20Health%20Issues%20White%20Paper.pdf, http://www.cdms.uci.edu/PDF/resilience-workshop.03222012.pdf, [10]).

Although the article describes many changes in the programs, many of these appear structural and locational. Beyond the description of use of CBT and non use of Benzodiazepines, it was not clear exactly what type of CBT is being offered, when it is offered, how providers are trained in these models, how this training is funded and if children are also being served with CBTs.

It is not clear exactly what type of CBT is being offered, how providers are trained in these models, how this training is funded and if children are being served. The range of services appears comprehensive, but the article is lacking in sufficient detail to permit further conclusions. For example, the word “resilience” is used frequently to describe program elements and in the “current model” it refers to “on-going efforts to promote public resilience during routine times through resilience centers and other NGOs”. However there is no specific operationalization provided on exactly what “efforts to promote public resilience” means, if it has evidence of effectiveness, and how it is delivered and to whom.

Further, care is described as being provide in a “stigma free” manner, but how is this determined? “Stigma free” is most likely a goal, not a current outcome. Rates of utilization, compared to population level estimates of new incidence or point prevalence of post disaster disorders are not presented, so it is not clear to what extent services are achieving population level “reach” to those at risk. Available epidemiological reviews suggest that between 30–40 % of the population can be at risk for a disorder following a disaster. Often times these outcomes reflect comorbid presentations of PTSD plus another disorder such as depression. However, most of the services seem directed to “anxiety” or distress, not clinical depression or PTSD. The latter can be part of the outcome trajectories for some persons experiencing trauma, including young children, as our diagnosistic systems increasingly recognizes changes in the expression of PTSD in children 6 and under (http://www.apa.org/pi/families/resources/task-force/child-trauma.aspx). In addition, there is a phenomenon that can be separate from anxiety disorders including PTSD and is known as traumatic grief [11–24]. In children, this can present similarly to PTSD but requires fundamental changes in the way care is provided. Since many of the real world events that Israel has experienced also involve traumatic loss of loved ones, it is not clear from the article to what extent a response to traumatic loss and grief have been included in services provided.

The foci of the evolution of services appear to be related to location of services, with the exception of the addition of tele-health services when the threat of injury while seeking mental health care became a further barrier. More information on these exciting efforts that are “resilience” focused, i.e. pre-event and “public” focused is needed. Indeed, emerging efforts in the United States and elsewhere have begun to focus on models of stepped care which involve early evidence-based mental health triage, brief acute phase intervention for those at risk, with symptoms and desiring care followed by reassessment and then provision of a full course of Trauma Focused CBT, in the case of children for example. In the disaster and terrorism situation, an example of this approach is the so-called “National Children’s Disaster Mental Health Concept of Operations [25–27]. Rather than a “one time, one size fits all” approach, this model allows for allocation of limited professional mental health resources to those at greatest risk using a “floating triage algorithm [28, 29]), provision of timely care (which has evidence of being differentially effective, [30, 31] and access to moderate or longer term care if still required to complete the stepped care continuum.

### Location of care

Given the unique nature of the threats encountered and the population distribution coupled with surge demands in hospital emergency departments, the Israeli emergency mental health program has evidenced great changes in locations of care—all informed by lessons learned from real world events. Innovations such as co-locating services with hospital emergency departments were attempted, but then modified or discontinued. Since the observation that many high-risk individuals are present in hospital ED’s, it is not clear whether the discontinuation of the use of these the modified ED sites has been beneficial. It is also unclear why ambulances were seemingly used to transport individuals with primary anxiety unless they also were physically injured and required ambulance transport. This may reflect Israel-specific cultural or organizational considerations.

Despite changes over time, the basic model seems to a variant of a stand-alone mental health clinic. referred to as “resilience centers” or by some other terms, they represent largely stand alone clinical settings. One wonders to what extent the inclusion of other settings such as primary care, schools, shelters, work settings and among responders of various types have been included and whether adequate attention has been given to coordination between these settings. Although tele-health is mentioned, other innovations in disaster mental health might be considered to further improve access to care and population level “reach”, which remains elusive in the US [32] and apparently in Israel as well. These include the use of Internet based interventions [33] and other interventions for definitive clinical depression and PTSD, which already exist. Provision of care either in the home via tele-health or Internet based interventions or in more routine “disaster systems of care” [26, 34] would further extend the reach that has been so challenging in disasters.

### Summary

The system of disaster mental health in Israel has demonstrated considerable growth and evolution in the last 25 years and reflects collaborations across many different levels and partners. At its most important, the article by Bodas et al. outlines a national commitment and recognition of the psychological impact of chronic conflict and terrorism. The emergency mental health program has evidenced multiple changes and demonstrates an aggressive “lessons learned” process. Challenges such as timing, service delivery settings, models of care, payor/funding and coordination issues were reviewed. As the program moves forward into the future, recommendations include broader inclusion of “disaster systems of care” [26, 27] to include re-engaging the emergency medical system and hospitals, schools, child care, disaster relief, first responders, and emergency management across all sectors of government. Recommendations also include an increasing focus on populations beyond an exclusive focus on symptomatic individuals, to sub-populations including all children impacted by conflict, those with prior trauma or mental health history, traumatic exposure, communities vs individuals, and responders broadly conceived. An adult and child emergency mental health CONOPS continuum of care approach, that better integrates preventative efforts, acute phase response coupled with rapid triage in primary touch points or disaster systems of care and access to evidence based treatments for adults and children [26, 27] is recommended for consideration, as the program moves forward into the future.
